# Coordination of human movements resulting in motor strategies exploited by skilled players during a throwing task

**DOI:** 10.1371/journal.pone.0223837

**Published:** 2019-10-17

**Authors:** Bao Nguyen Tran, Shiro Yano, Toshiyuki Kondo

**Affiliations:** Department of Computer and Information Sciences, Tokyo University of Agriculture and Technology, Koganei, Tokyo, Japan; University of Texas at San Antonio, UNITED STATES

## Abstract

In this study, we investigated the underlying mechanisms of a motor system that affects skills and strategies of expert dart throwers. Eight experts participated in our experiment and each subject performed 42 throws. Kinematics of the shoulder, elbow, wrist, and dart were recorded by six high-speed cameras (200 Hz). The vertical error curve over time was calculated based on both hand and dart trajectories to clarify their relationship and interaction, which could attribute to their skills. Moreover, the kinematics of the dart (speed and direction) and angular kinematics of the elbow and wrist at the time of release were investigated to elucidate which parameters constitute the throwing strategies of experts. Experimental results showed that expert’s throwing can be classified into two strategies, i.e., reducing timing sensitivity and reducing timing error. These strategies were derived from the spatial and temporal controls of the hand trajectory. Moreover, we confirmed that the speed of the dart and angular acceleration of the wrist joint at the time of release were highly correlated with the time-window for successful release. These results imply that the two strategies are characterized not only by a spatiotemporal relationship between the hand and dart trajectories, but also by relationships with release kinematic parameters of the proximal joint and the dart. Understanding characteristics which lead to strategies of skilled throwers would provide effective training methodology for beginners.

## Introduction

The ability to grip and throw an object has been considered a survival advantage of human beings in the early stages of evolution to hunt and defend against predators [1,2]. The motion at the wrist has been investigated for several clinical and sports applications [3–6]. Accurate throwing becomes a critical skill in modern sports such as baseball, basketball, handball, and dart throwing. Therefore, it is important to understand the underlying mechanisms of the motor system to improve performance and train novices. In this study, we investigated which kinematic parameters and patterns from hand trajectory exploited by experts in dart throwing lead to their throwing strategies.

In any throwing movement, the initial values of position, speed, and direction of the projectile directly determine the accuracy of a throw. These parameters are provided by the angular kinematics of main articulations of the upper limb (shoulder, elbow, and wrist) at the time of release. As a result, timing precision may be a critical factor contributing to the accuracy of a throw. Several studies of throwing movement have *separately* investigated the influence of either kinematics of joint movements or the projectile at the time of release during a performance.

With regard to the kinematics of human joints, Hore et al. found that the onset time of finger extension [7] or the variability of the time of release [8] highly affected the performance in ball throwing. In basketball free throwing, Verhoeven et al. [9] found that releasing balls closer to the moment at which the center-of-mass reached peak height was considered a skill. In javelin, the angular speed of the shoulder girdle and forearm had a high correlation with the measured distance [10]. In addition, Toffan et al. [11] indicated that the position of the back foot at the time of release had a strong effect on throwing test score in football-quarterback throwing. However, it is insufficient to evaluate only one release parameter due to the redundancy problem [12]. In addition, variance in one parameter of a joint could be compensated for by variance in a parameter of another joint to achieve accurate throws [13]. Importantly, even though kinematic patterns of human joints at the release moment can be an indicator to classify levels of throwers [10,13–16], the relationship between those factors and strategies among experts is still ambiguous.

Considering the projectile’s parameters, in some sports where throwing distance is the key performance indicator such as javelin or boccia, the release speed individually affects the outcome [10,14,17]. However, in the case of goal-oriented throwing tasks, there are various combinations of position, speed, and direction which could result in the same final position. Moreover, Kudo et al. [18] found that release parameters could co-vary without influencing the outcome. Furthermore, several studies predicted the final position of a projectile as well as investigated timing sensitivity by prediction models based on the hand trajectory [19–22]. In these studies, it was assumed that the hand moved exactly as the projectile does at the release moment. Smeets et al. [19] determined the timing sensitivity through the movement of the thumb’s kinematics and found that better performance is not a result of trajectory adjustments which reduce the timing sensitivity of release. However, it could be more precise and reduce the complexity of mathematical problems if we captured the projectile trajectory directly. Cohen et al. [20] proposed a new approach that calculates an error curve derived from the hand trajectory in the virtual throwing task. After practice, skilled throwers in their experiment increased the time-window for successful release and reduced timing error. However, the task was implemented virtually, which differed from a real throwing task with many degrees of freedom and constraints. Using a similar method, Nasu et al. [21] found that experts exploited the two strategies in dart throwing, i.e., reducing timing sensitivity and reducing timing error, based on error curves derived from the index finger tip trajectory. However, the prediction model might be imprecise because of the difference between the trajectories of the index tip and dart during the acceleration phase of a throw. Indeed, when the hand moves closer to the release moment, the two trajectories become different since the index tip starts to move laterally to release, whereas the dart continues to go straight forward. Furthermore, since the relative position between the index finger and a dart is significantly dependent on gripping ways of throwers, this prediction model cannot be generalized. As a result, the model in this study estimates the vertical error based on measured trajectory of the projectile, thus it should be more precise.

In brief, neither human postural control nor the projectile movement at the time of release can represent the overall skills or strategies of individuals. Few known studies investigated the relationship between these parameters and the strategies of skilled throwers. Therefore, this study aims to clarify how experts control the angular kinematics of proximal joints (elbow and wrist), hand trajectory, and projectile’s parameters (speed and direction), and connect these factors with throwing skills and strategies in dart throwing. Our hypotheses were as follows: 1) Experts who reduce timing sensitivity might pre-plan to optimize the hand trajectory before the time of release, which implies that these throwers might have spatial control and less focus on timing precision. Meanwhile, hand paths of experts who reduced timing error are not optimized. 2) Kinematic parameters from proximal joints (i.e., the angular acceleration of the elbow and wrist) and the dart (release speed and direction) would affect the timing sensitivity, reflecting specific features of each strategy. Therefore, this implies that the strategies are not only characterized by the hand trajectory as reported in previous studies [20,21].

## Materials and methods

### Subjects and ethics

Eight neurologically healthy and right-handed dart players (6 males and 2 females; height: 177.25 ± 9.07 cm) participated in our experiment. Their handedness was confirmed by using the Edinburgh Handedness Inventory [23]. All participants had been competitive players and dart players for an average of 9.0 ± 4.60 years. Their best recorded scores in a count-up game ranged from 1083 to 1210. The maximum score in one count-up game is 1440 (24 throws with a maximum score of 60 for one throw). The subjects were informed about the procedure and provided written informed consent prior to the experiment. The experimental procedures were approved by the ethics committee of the Tokyo University of Agriculture and Technology.

### Experimental setup

In our experiment, hard darts (steel tip) were used, and all subjects confirmed that they could adapt to the darts. The center-of-mass (COM) was determined beforehand. Participants were asked to stand in front of the throwing line (237 cm away from the dartboard) and aimed for the bull’s-eye, which is 172 cm off the ground. Although we simultaneously recorded data of both kinematics and electromyography (EMG) in this experiment, we only focused on the kinematics in the following analysis.

We utilized six infrared cameras (Prime 13, Optitrack, Inc.) for motion capturing and used one of these cameras in MJPEG mode to detect an LED light for trigger (explained below) and record the throwing motion. All cameras were located appropriately, so they could capture movements of the subject while throwing and the movements and trajectory of the dart during flight. We calibrated and adjusted the workspace display on a PC before the experiment, and the overall re-projection error of the calibration was 0.82 ± 0.02 mm. Thereafter, we created a coordinate system for the experiment. For EMG recording, WEB-5000 system (Nihon Kohden, Japan) was used and connected to the PC through a National Instruments data acquisition device (NI-USB 6218 BNC). Muscular activities of subjects were recorded by a wireless device that was connected to eight surface electrodes attached on eight muscles along the arm. Additionally, the trigger signal from the LED light was fed to the data acquisition device with the same recording rate as EMG data.

### Procedure

The experiment took approximately an hour and was divided into four stages: *warm up*, *preparation*, *confirmation*, and *throwing*. Throughout the experiment, we recorded EMG and kinematic data per set, which included six throws. After one throw, the dart was taken out of the board immediately to prevent it from being overlapped or hidden by the next dart’s final position. Subjects started each set of throwing when given a verbal command by an experimenter.

*Warm up* stage: Before the experiment, all subjects were asked to practice for three minutes to get used to the darts, which was followed by a *baseline* session with 18 throws (three sets of six throws). Thereafter, subjects were asked to sit so that the experimenter could attach the electrodes and markers for the next stage.

**Fig 1:**
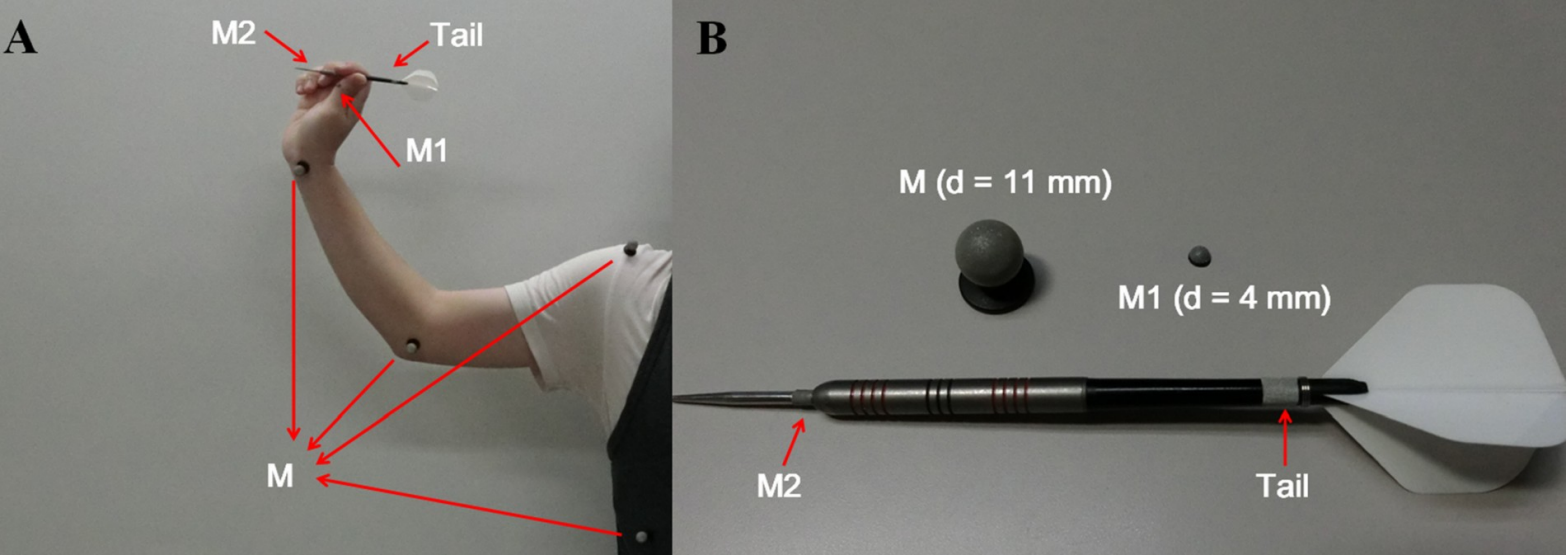
Markers used in this experiment. (A) Markers attached on various parts of the arms of the participants and on the dart during throwing. (B) Marker sizes.

*Preparation* stage: For tracking the movements of the trunk, shoulder, elbow, and wrist joints, we attached four hemispherical facial markers (M markers in Fig 1A) on the corresponding anatomical landmarks: *7*^*th*^ *rib*, *acromion process*, *medial epicondyle*, and *distal radioulnar joint*. A smaller marker (d = 4 mm) was attached on the metacarpophalangeal joint of the thumb (referred to as *M1* in Fig 1A and Fig 1B). Moreover, we utilized two reflective tapes attached on darts to capture dart movement. The first tape was wrapped around the dart’s tip and was 20 mm away from the COM (near the dart tip) to create a marker (M2), and the second one was attached on the bottom of the dart shaft (“*Tail*” in Fig 1A and Fig 1B). We assumed that the dart’s final position was the same as the position of marker M2. EMG and kinematic data were recorded at 2000 Hz and 200 Hz, respectively, and we synchronized the two kinds of data by the trigger signal. That is, the moment (frame) at which the LED light changed from OFF to ON, as observed by the camera in MJPEG mode, was synchronized with the sample of the trigger signal when it changed from high voltage to low voltage.

*Confirmation* stage: After wearing EMG electrodes and attaching the markers (described above), subjects were asked to perform 18 throws (three sets of six throws) in a *post* session to confirm whether discomfort or any other inconvenience affected their performance. To evaluate the effect, we compared the performance differences between the baseline and post sessions (see “Performance evaluation”).

*Throwing* stage: Subjects performed 42 throws (seven sets of six throws). In this study, only kinematic data of these seven sets were analyzed.

### Detecting the release moment

Since the distance between the thumb and dart tip was almost unchanged during the throwing period and suddenly increased at the release moment, we determined the release moment by the two following conditions. First, the release moment was detected when the Euclidean distance between markers M1 and M2 at this moment exceeded a threshold, which was set to the maximum distance measured within the first 150 ms when subjects started to cock back the forearm. Second, the difference between the two distances at the next two frames (5 ms) after the moment detected by the first condition was larger than 25 mm. The second condition ensured that the dart was released because, in rare cases, the distance slightly fluctuated (up and down) around the threshold when the arm moved in the acceleration phase, which leads to false detections. We also utilized the camera data recorded in MJPEG mode to check the time of release.

### Data processing

All kinematic data of the human arm and dart were low-pass filtered at 20 Hz, using a zero-phase-lag 2nd-order Butterworth filter. After detecting the release moment of a throw (see “Detecting the release moment”), to obtain the precision of 1 ms and make the data more easily comparable to data reported in previous studies [20,21], we interpolated the data into 1000 Hz using a cubic spline interpolation. Data analysis and statistical test were performed using MATLAB (Mathworks Inc.).

### Performance evaluation

To evaluate the performance, we first determined the target coordinates in the coordinate system. Before the experiment, we put a hemisphere facial marker (d = 4 mm) on the center of the bull’s-eye of the board and measured its location (referred to as *target position*). Afterwards, we removed the marker. During the experiment, after a throw, we measured the final dart’s position by marker M2 on the dartboard. The vertical and horizontal errors of the throw were calculated by taking the difference between the two positions, i.e., *target position* and marker M2 on the board. In the horizontal plane, the dart’s final position is only determined by its initial position and release direction since there are no forces acting upon the dart after the time of release [19]. Due to the simple linear relationship in controlling horizontal error, we only focus on the vertical error on the board.

In further timing sensitivity analysis, we only utilized the data of successful throwing, which was defined that the resultant dart position is within the bull (i.e., the outer circle of bull’s eye with 16 mm radius). In other words, a throw was considered successful if the absolute vertical error was less than 16 mm. The performance of the experts was evaluated by the absolute vertical error and the success rate (out of 42 throws).

To check whether the discomfort due to the electrodes influenced the performance on the board, we used the Student t-test to evaluate the difference of the absolute of the vertical error between the *baseline* (18 throws) and *post* sessions (18 throws) within a subject.

### Time series of vertical error based on dart tip movement

We assume that the COM of the dart moved exactly the same as that of marker M2 at the release moment and then flew with a parabolic trajectory after the moment, neglecting air resistance and rotation. Vertical error at time t was calculated according to the following equation [20,21]:

$${Ey}_{t}=\left(y_{t}+tan\theta_{t}\times {{(x}_{TG}-x}_{t})-\frac{9.8}{2}\times \frac{{\left(x_{TG}-x_{t}\right)}^{2}}{{\left(v_{t}\times cos\theta_{t}\right)}^{2}}\right)-y_{TG},$$

where *x*_*TG*_ and *y*_*TG*_ are the horizontal and vertical coordinates of the *target position*, respectively; *x*_*t*_, *y*_*t*_, *v*_*t*_, and *θ_t_* are the horizontal and vertical coordinates, speed, and direction of the dart (magnitude and angle of the dart-release vector, respectively) at time *t*, respectively.

In each throw, there was always an estimated error (*offset*) between the calculated location at the release moment and the final location of the dart measured by the motion capture system. To eliminate the estimated error, in each throw, we shifted the curve by adding or subtracting the *offset* value. In other words, for practicality, the calculated value after offset corrections at the release moment of the error curve was the same as the actual error on the board measured by the capture motion system.

In this study, since the actual impact position of a dart on the board was measured by the motion capture system together with the calculated position (*before offset corrections*), we evaluated this prediction model by the following indices: (1) the absolute of the estimated error, (2) the average of correlations between the actual and calculated positions across all experts, and (3) the correlation between the standard deviations of the actual error (measured by the motion capture system) and calculated error among all experts.

**Fig 2:**
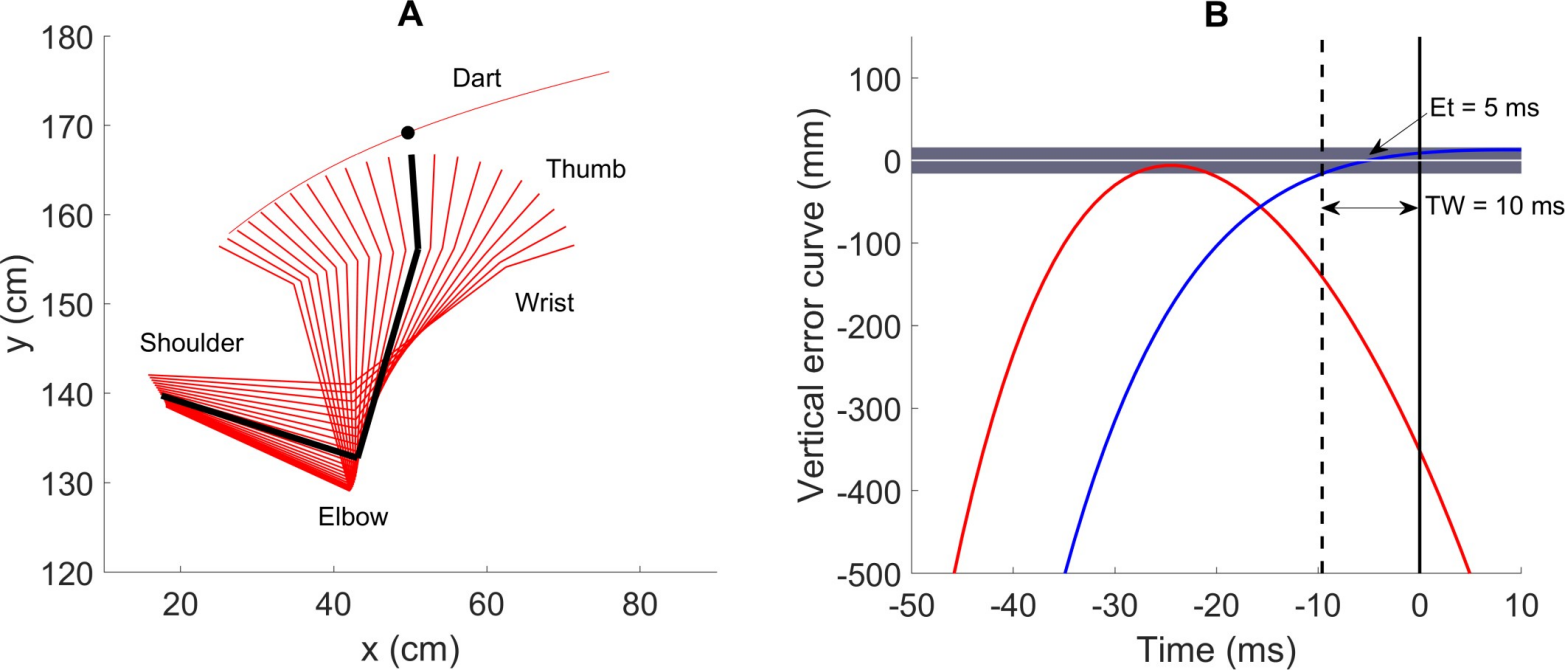
Vertical error curve derived from the thumb and dart movement for a single throw. (A) An example of the coordinate data in the sagittal plane of the dart trajectory and human joints recorded during a throw (during a 105-ms time-window started from 60 ms before the release moment, at 5-ms intervals). The red curve represents the dart trajectory. The red line describes a segment between two joints, and every three connected red lines indicate the position of the throwing arm at a certain time. The thick black lines and black round marker describe the position of the throwing arm and the dart position at the release moment, respectively. (B) Vertical error curves were derived from the thumb (red) and dart (blue) trajectories. The black line represents the release moment. The hit zone and the zero line (optimized error) are represented in the dark gray zone and horizontal white line, respectively. The dashed black line demonstrates the moment that the blue curve crossed the hit zone and the time-window (TW) in which the thrower can release for an accurate throw (10 ms in this example). The timing error (Et) is 5 ms, determined by the absolute timing difference between the actual and optimal timing (zero cross line or minimum error value).

We also calculated the vertical error curve derived from the thumb movement by the same equation with the dart, but with the kinematic parameters of marker M1 at time t. The vertical error curve derived from the thumb was *not* calculated to predict the actual final position of the dart on the board because of a large estimated error [19]. Instead, it was used to investigate the relationship between the thumb and dart trajectories temporally and spatially. Moreover, a Wilcoxon signed-rank test was conducted to assess the difference between the estimated errors calculated from dart marker (M2) and finger marker (M1) trajectories to confirm that our model based on the former can estimate the final position more precise. Fig 2A shows movements of the throwing arm (stick figures represent in red lines) and the dart trajectory (red curve) during a successful throw. From the coordinate data of the throw in Fig 2A, the corresponding vertical error curves derived from the thumb and dart trajectories are described in Fig 2B.

### Time-window length for successful throwing and timing error

In order to evaluate the throwing quantitatively, we introduced two indexes: time-window length (TWL) and timing error (Et).

We defined the time-window length for successful release as the absolute time difference between the moment at which the vertical error curve derived from the dart entered the hit zone and the release moment (Fig 2B). In each subject, TWL was calculated and averaged within the successful throws. Although a TWL for the successful release can be considered as the length of an ideal time range where all single release moments could result in a successful throw, the current definition results in an under-estimation of the ideal TWL since we did not include the period after the time of release.

We measured timing error (Et) within successful throws based on a previous study [20], by taking the absolute of the timing difference between the actual and optimal release moments. The latter is determined by the moment that the blue curve crossed the zero line (Fig 2B). In some throws, if the error curve did not cross the zero line, the optimal release would be determined by the moment when the curve got the smallest value within 10 ms after the time of release. The data of the curve later than 11 ms after the release moment were eliminated since the dart at that time flew far away in that period.

In addition, the variability of release times (VRT) was calculated by the standard deviation of release moments relative to the zenith of the hand path to investigate whether stabilizing the release times was used to compensate for high timing sensitivity, as reported in a previous study [21].

### Correlation between hand trajectory and the strategies

To test the first hypothesis, which investigates the relationship between the hand trajectory and throwing strategies, first, we calculated the average of the peak values (spatially) and peak times relative to the release moment (temporally) of the error curves derived from the thumb (the red curve in Fig 2B). Next, the relationship was evaluated by the correlations between these two parameters and TWL. Moreover, we investigated whether the stability of the hand trajectory is correlated with strategies through the relationship between the standard deviations of these two factors and TWL.

### Correlation between kinematic parameters and the strategies

To realize accurate throw, skilled throwers have to control complex combinations between the proximal joints (elbow and wrist) and hand trajectory and release the dart at an appropriate speed and direction. Consequently, the strategies of experts related to timing sensitivity might be caused not only by the hand trajectory as suggested by previous studies [20,21], but by other kinematic parameters of proximal joints and the dart. That is, angular velocity, acceleration, and jerk of the elbow and wrist joints, and speed and direction of the dart at the release moment. Therefore, correlations between these parameters and TWL were investigated in this study.

## Results

### Influences of wearing devices and markers

The vertical error was not significantly different between the *baseline* and *post* sessions (*p* > 0.05, all, Table 1), which means experts have quickly adapted to the discomfort caused by attaching the electrodes and markers during the *post* session. Importantly, there was no effect of the discomfort on the following *throwing* stage, which was the main experiment. There was a possibility that by learning, the experts compensated for their performance degradation during the *post* session. However, the possibility was very low, because the number of throws is only 18, and it is natural to consider that the effect of attaching electrodes on the motor skill of experts is quite limited.

**Table 1:** Performance of each subject.

Subject	*p value*	Success rate (%)	Absolute vertical error (mm)	SD of vertical error (mm)
1	0.85	57.14	13.12	10.17
2	0.28	64.29	12.75	10.43
3	0.15	54.76	15.36	9.54
4	0.80	69.05	13.20	11.02
5	0.92	52.38	16.83	14.41
6	0.87	69.05	14.25	11.13
7	0.52	52.38	18.17	15.63
8	0.51	57.14	15.48	11.58

SD: standard deviation

### Performance on the board

Table 1 shows the successful throwing rate and the absolute of vertical error for all subjects. The former ranged from 52.38% to 69.05%, and the latter ranged from 12.75 ± 10.43 mm. Subjects 5 and 7 exhibited the smallest success rate (52.38%) and the largest vertical error (mean value > 16 mm), whereas subjects 2 and 4 could be considered the best performers.

### Validation of the prediction model and time series of the vertical error curve

**Fig 3:**
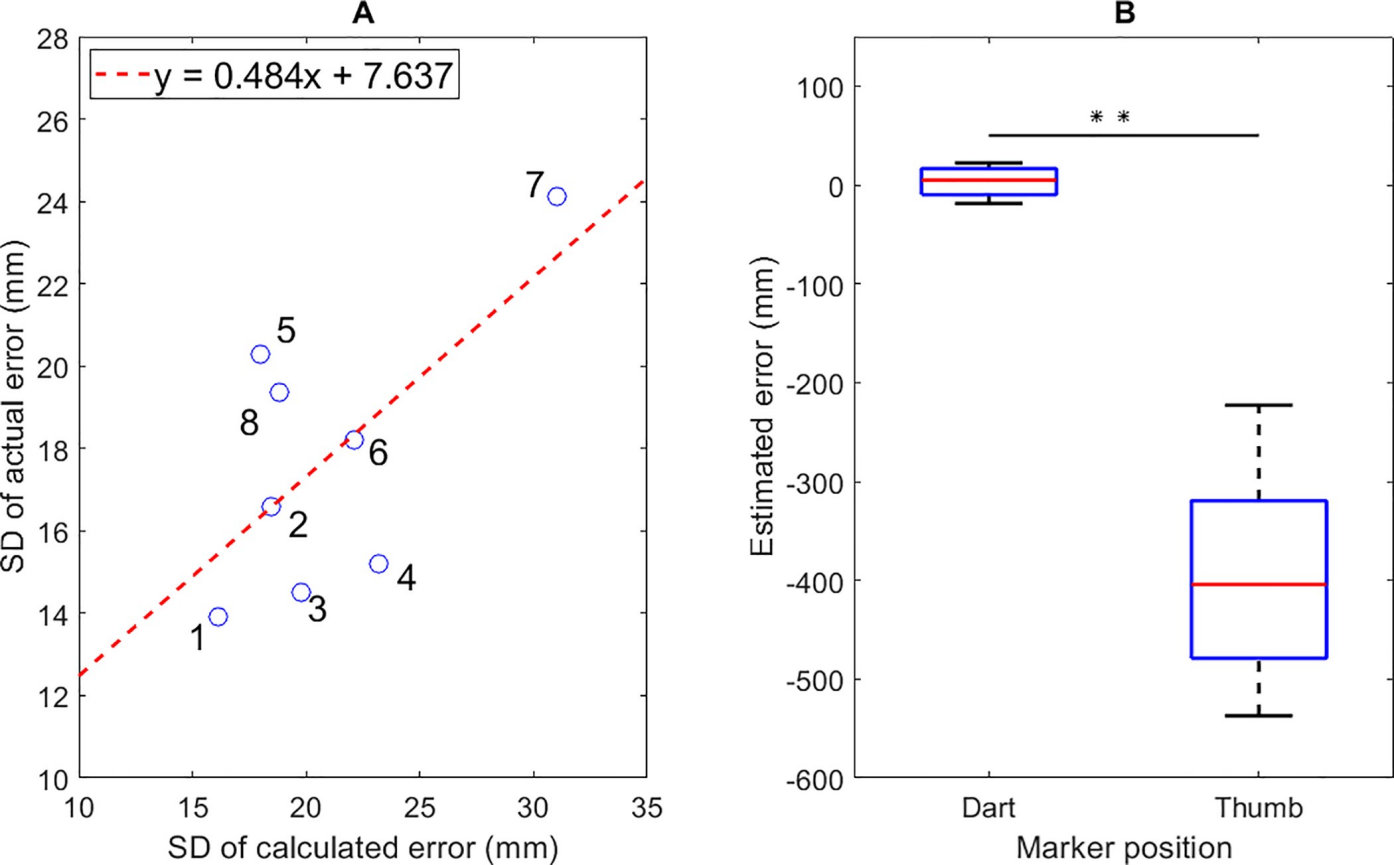
Validation of the prediction model in this study. (A) Standard deviations (SD) of the actual error and calculated error. The dashed red line represents the linear regression line. (B) Estimated errors calculated from the dart and finger trajectories. **: *p* < 0.01.

For validation of the prediction model, we examined the three indices mentioned above (see “Time series of vertical error based on dart tip movement”). In this study, all the absolute estimated errors were not larger than 40.0 mm, with an average of 14.89 ± 5.0 mm among the subjects. Furthermore, a high correlation was found between the calculated and actual positions on the board (*r* = 0.86 ± 0.05 among subjects). Moreover, Fig 3A shows the correlation between the standard deviations of the actual error and calculated error (*r* = 0.65, *p* = 0.08). From the results of the three indices, the model can reasonably estimate the measurement. Furthermore, there was a significant difference between the estimated errors calculated from dart trajectories (3.30 ± 15.67mm) compared to the ones calculated from finger trajectories (-395.66 ± 109.38 mm); Z = 36, *p* < 0.01 (Fig 3B). The result suggested that the final resultant position of the dart estimated based on its trajectories could be more precise than that estimated from the finger trajectories as in a previous study [19].

**Fig 4:**
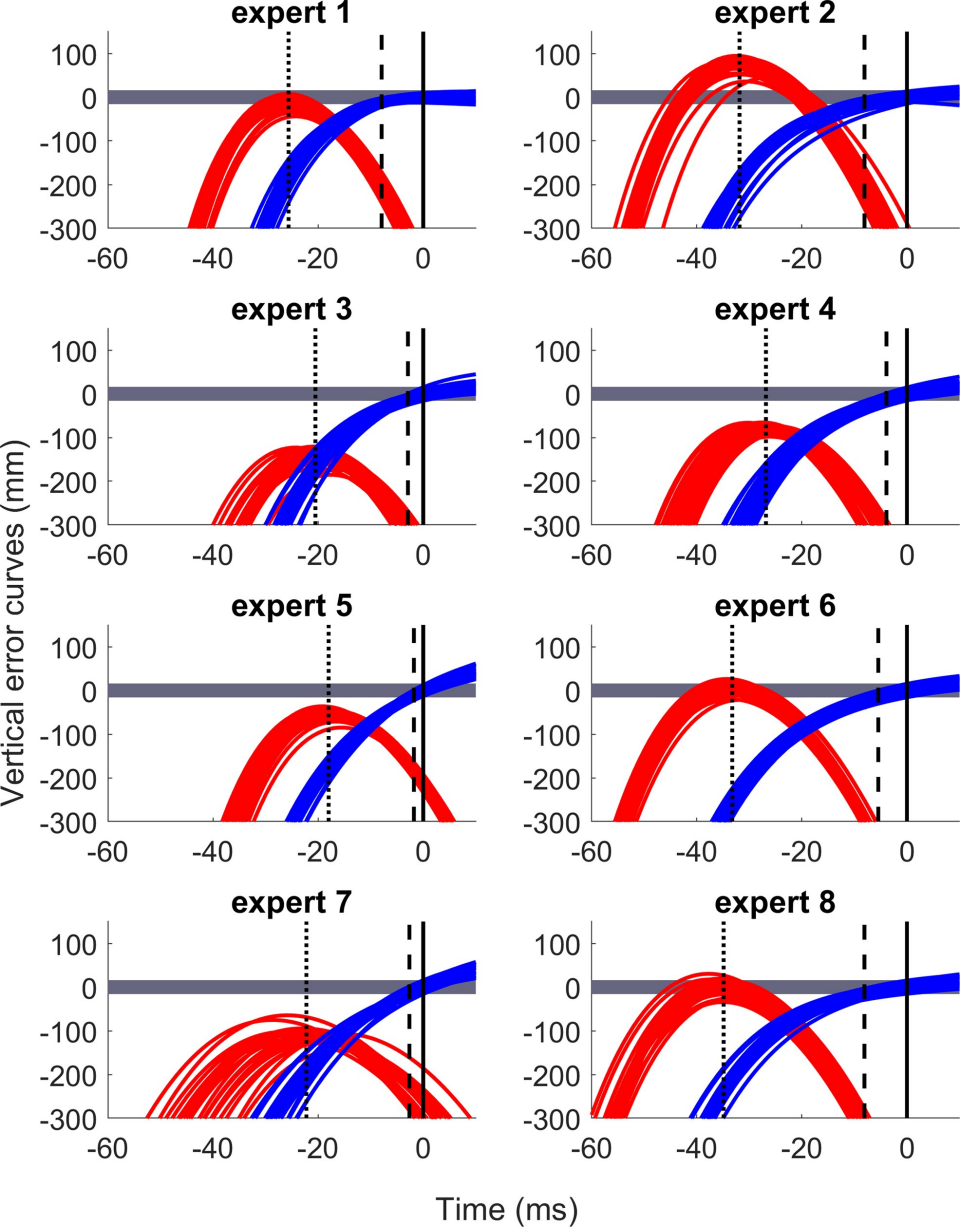
Time series of vertical error curves for all experts. Red lines represent the vertical error curves derived from hand trajectory. Blue lines indicate the vertical error curves derived from dart trajectory. Black lines demonstrate the release moment. Dotted black lines indicate the average timing at the peak value of red curves relative to the release moment. Dashed back lines represent the average TWL relative to release moment. The hit zone is displayed in dark gray.

Fig 4 illustrates the patterns of vertical error curves derived from hand and dart trajectories (markers M1 and M2, respectively) for successful throws. The curves derived from dart’s movements crossed the hit zone only once because the values at the time of release were equal to the vertical errors measured on the board. The benefit of this method is that the error curve derived from dart movement crossed the successful zone only once before the time of release, which could be more explicit and intuitive to evaluate the TWL of a throw (Fig 4). Moreover, the point of optimal release was reduced to one instead of two, as compared to the previous models.

**Fig 5:**
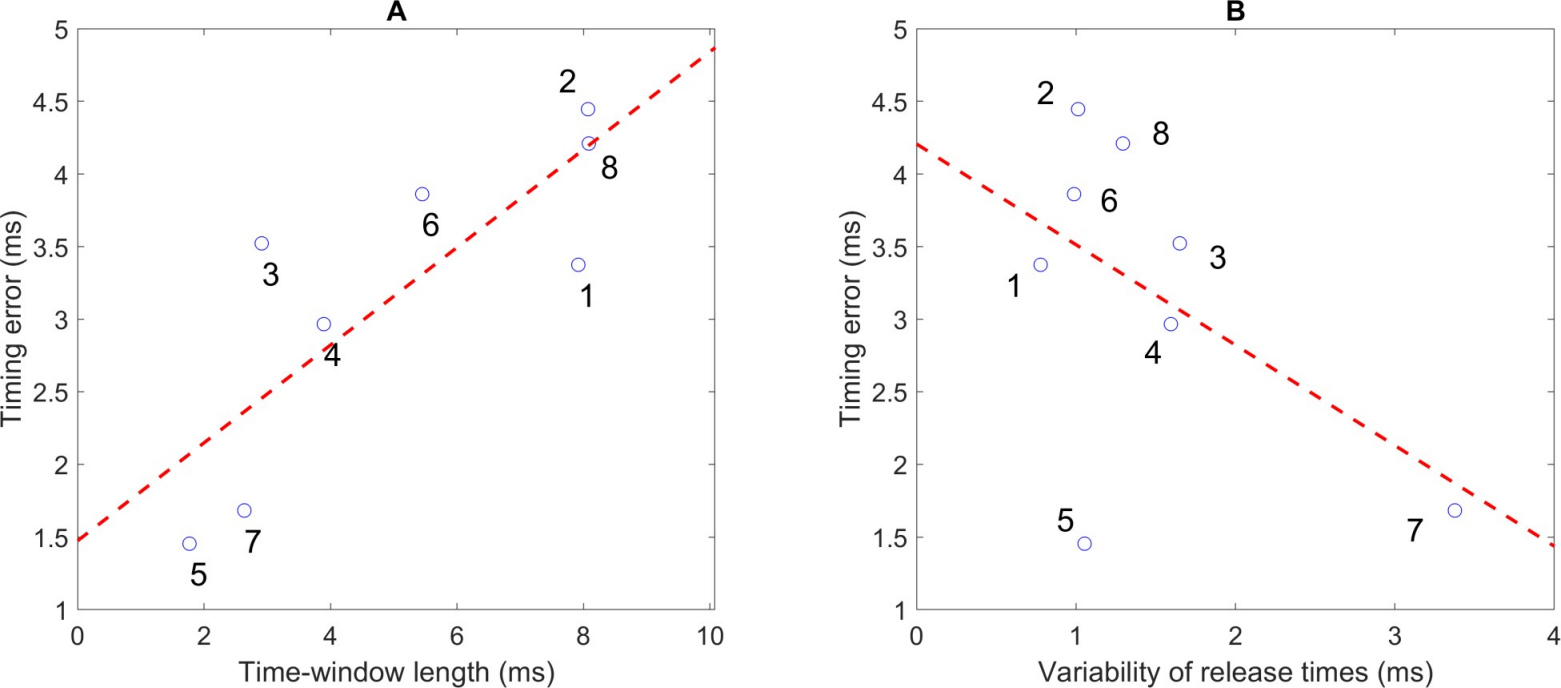
Relationships between timing parameters reflecting strategies of the experts. (A) Relationship between time-window length (TWL) and timing error (Et) of the experts. (B) Relationship between Et and the variability of release times relative to the zenith of the hand.

Fig 5A shows the relationship between TWL and Et across the experts, with a high correlation (*r* = 0.81, *p* = 0.02). Based on TWLs, we divided the subjects into two groups: *longer* group (subjects 1, 2, 6, and 8), with TWLs that ranged from 5.45 ms to 8.08 ms; and *shorter* group (remaining subjects), with TWLs that ranged from 1.77 ms to 3.90 ms. In this study, the Et of the experts ranged from 1.45 to 4.44 ms. Fig 5B shows the correlation between Et and the variability of release times (VRT) relative to the zenith of the hand, which is quite low (*r* = -0.52, *p* = 0.19). Seven of eight experts performed the task similarly with very small VRT (ranged from 0.78 ms to 1.65 ms), except expert 7 (3.38 ms).

With regard to the time series error curves derived from thumb movement, most of the curves in the *longer* group crossed or peaked very close to the hit zone. Meanwhile, the peaks of the curves in the *shorter* group did not reach the zone, with large error values before the release moment. In terms of timing, there was a difference in the peak times relative to the time of release between the two groups, with about 32 ms in the *longer* group and 20 ms in the *shorter* group. Exceptions were experts 1 and 4, who had a similar timing of 26.83 ± 1.69 ms, respectively, although they belonged to different groups (see Fig 4).

### Relationship between the hand trajectory and the two strategies

**Fig 6:**
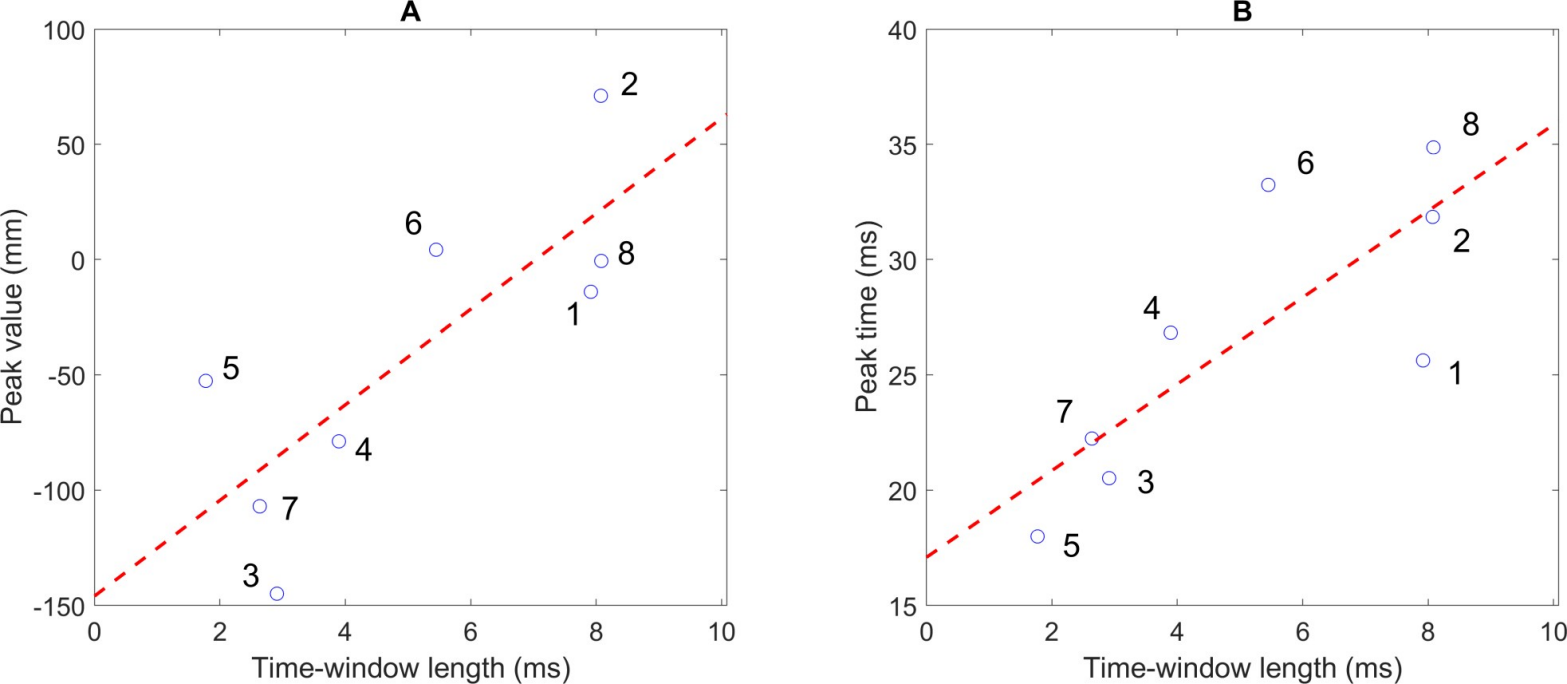
Relationship between hand trajectory and the strategies. (A) Correlation between peak value and time-window length (TWL) for successful throws. (B) Correlation between peak time relative to the release moment and TWL for successful throws. In both figures, the dashed red line represents the linear regression line.

To clarify whether kinematic parameters of the hand trajectory were correlated with the strategies, we evaluated the correlations between the average of peak values and peak times (relative to the release moment) of the curves derived from the thumb and TWL. As shown in Fig 6, we found a significantly strong correlation between the means of the two parameters of the error curves from the thumb and TWL, with *r* = 0.79, *p* = 0.02 at peak value and *r* = 0.80, *p* = 0.02 at peak time. This result suggests that the strategies of experts characterized by the TWL were correlated with kinematic parameters of the hand trajectory in both spatial and temporal domains. However, we did not find a strong correlation between the standard deviations of the two parameters and TWL, with *r* = 0.09 at peak value and *r* = -0.44 at peak time.

### Kinematic parameters of the dart and human effects on the two strategies

**Fig 7:**
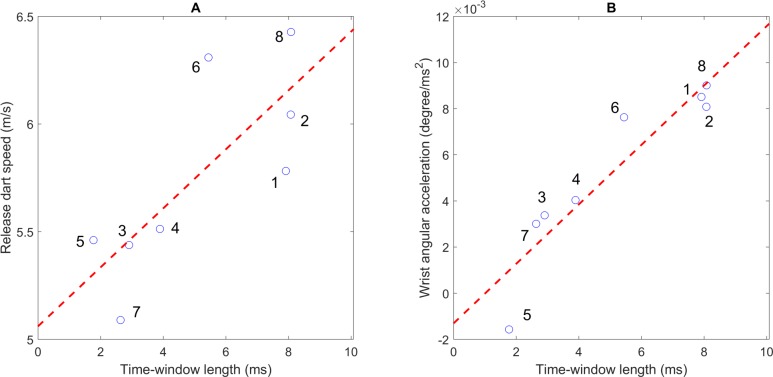
Relationship between kinematic parameters and the strategies. (A) Relationship between the speed of dart at the time of release and time-window length (TWL). (B) Relationship between angular acceleration of the wrist and TWL. In both figures, the dashed red line represents the linear regression line.

As shown in Fig 7A, we found strong correlations between the dart’s speed at the time of release and the TWL across all experts (*r* = 0.78, *p* = 0.02), suggesting that throwing at a high speed leads to a longer time-window for a successful release. We also found that the larger release speed was associated with a smaller release angle of the dart to obtain the accuracy (*r* = -0.86, *p* = 0.006) across eight subjects, which reflects the strategies of the experts. Similarly, we found that wrist angular acceleration was highly correlated with TWL (*r* = 0.93, *p* = 0.0009, Fig 7B). However, we did not find any correlations in kinematic parameters at the elbow joint.

## Discussion

The results of our study showed that expert’s throwing can be classified into two strategies, i.e., reducing timing sensitivity and reducing timing error. These strategies were derived from the spatial and temporal controls of the hand trajectory. In addition, we confirmed that the speed of the dart and angular acceleration of the wrist joint at the time of release were highly correlated with TWL for successful release.

### Confirming the two strategies

The mean value of Et and the correlation trend were similar to a previous study in dart throwing [21], but only one expert showed a much longer TWL (12.8 mm) in that study. In addition, longer TWL was associated with larger Et (*r* = 0.81, Fig 5A) among all subjects, which confirms that there were two strategies in experts: reducing timing sensitivity and reducing timing error.

In the current study, the correlation between Et and VRT was low (*r* = -0.52, Fig 5B), which means that stability in the release times was not used to compensate for high timing sensitivity. We did not reduplicate the high correlation (*r* = 0.93) as in a study [21] because of the following reasons. First, experts 1, 2, and 6 (*longer* group) performed the task with the smallest VRTs, which means that very skilled throwers with low timing sensitivity could also stably control the release relative to the zenith of the hand path. Second, using the zenith of the hand as a reference to evaluate VRT might be imprecise since the kinematic landmark is also variable in the hand trajectory [19]. In other words, a lower variability of release times is not likely utilized to compensate for a shorter TWL as reported in [21].

### Hypothesis 1

To clarify whether experts might utilize the optimized hand trajectory in the acceleration phase to reduce timing sensitivity, we examined the relationship between kinematic parameters of the hand trajectory and the TWL.

We found high correlations between the peak values (*r* = 0.79) and peak times (*r* = 0.80) of the error curve derived from the thumb movement and the TWL (Fig 6). The experts with the reducing timing sensitivity strategy might pre-plan the optimized hand trajectory so that the corresponding error curve derived from the thumb crosses the hit zone about 30 ms relative to the release moment (see red curves in Fig 4). Afterwards, the throwers kept the direction of dart suitably (e.g., low-released direction) and drove the proximal joints forward at a particular speed for a successful throw. It could be considered a skill in spatial control with less focus on timing precision. This result aligns with that of a previous study [20], which reported that the key of this strategy is that the hand trajectory can be planned beforehand and does not rely on feedback of the previous throws. With regard to throwing tasks, previous studies discussed “modifying the hand trajectory” to increase the TWL as a strategy [21] or a training effect [20], but the particular pattern of this modification or improvement was unclear. In our approach, we have clarified the point, but have analyzed the interaction between the hand and projectile trajectory.

In contrast, the hand trajectory of the throwers in the *shorter* group was not optimized or all error curves did not cross the hit zone. Moreover, the peak was closer to the time of release, which caused them to choose the release moment more strictly (Fig 4). One could hypothesize that the hand trajectory of these experts was less variable to achieve accuracy within a shorter time-window. However, the correlations between the variability of kinematic parameters of the hand trajectory and TWL were not high: *r* = 0.09 (peak value) and *r* = -0.44 (peak time relative to the release moment). Especially, expert 7 had large variability in both peak value and peak time, but the subject had a very narrow time-window. This result indicates that the variability of the hand trajectory might not be representative of the throwing strategies, or experts with shorter TWL did not necessarily have hand trajectories that were more stable and stereotyped than the *longer* group. Instead, their strategy might be based on their practice of timing precision or other stable kinematic parameters.

### Hypothesis 2

Dart throwing is a sophisticated skill requiring several combinations of skills, such as forearm extension, wrist flexion, and release the projectile with appreciable speed, and direction to hit a very small target. In our experiment, the target’s diameter is just 32 mm. As a result, tiny displacements and variability in various parameters during throwing such as vertical lift of the shoulder, elbow, or dart at the release moment can significantly influence the performance, even though experts could compensate for these variabilities [13,18]. Thus, kinematics of the proximal joints (elbow and wrist) hardly influence the performance directly of an expert during a throwing task. However, if considering average values, we could analyze which parameters contribute to the different strategies among different experts.

We found that the TWL of experts increased as the angular acceleration of the wrist and dart’s speed at the time of release increased, with strong correlations (*r* = 0.93, *p* = 0.0009 and r = 0.78, p = 0.02, respectively, Fig 7) across all eight subjects. These strong correlations support the first hypothesis. If experts released a dart with a high speed, the throwers could release sooner or later since the dart flew straighter and was less affected by gravity. With such a high speed and a wrist angular acceleration during elbow extension, these experts might have to optimize the hand trajectory soon, about 30 ms before the release moment (Fig 4). Otherwise, the later the peak was reached, the larger the timing sensitivity became, and it was too difficult for the thrower to release at the “right” moment with the appropriately fast dart speed and wrist angular acceleration.

Conversely, in the *shorter* group, the experts released a dart with a lower speed, resulting in a high throwing arc to compensate, as the projectile was strongly affected by the gravity. Therefore, with high timing sensitivity, these experts might accelerate the wrist joint slower for easier release at an appropriate moment and do not need to realize the optimized hand trajectory. The results also imply that in dart throwing, the wrist joint, which directly grips and transfers the speed to dart, might have a more significant contribution in the skills of experts, rather than the farther proximal joint (elbow).

One could hypothesize that taller dart players chose the low arc (high-released speed) since the release vertical location of the dart was closer to the height of the bull’s-eye. However, in the cases of experts 2, 3, 4, 6, and 8, heights were similar (from 182 to 187 cm), but their strategies were different; additionally, this was true for experts 1 and 7 (170 cm in height). This implies that the strategy of low or high arc (long or short TWL) depends on the preference of players.

### The two strategies in a training perspective

In training, reducing timing sensitivity with spatial control would be easier for beginners. It is too difficult for inexperienced throwers to realize and throw with a TWL between 1.77 ms to 3.9 ms, like the experts in this study. Further, it is also difficult for them to have the ability to compensate for the variability within such a low time-window. Instead, by separating the two error curves derived from the hand and dart, the novices can first practice improving the wrist’s strength to accelerate faster and optimize the hand path based on the feedback of the derived error curves as the *longer* group, regardless of the error on the dartboard. After getting a good hand trajectory, the beginners will practice control and release the dart with appropriate speed and direction.

This study has some limitations. First, the sample size in this study (eight subjects) might not be sufficient to do correlation analysis among subjects, even though high correlations were found. Second, since the target was unchanged throughout the experiment (bull’s-eye) and the two best performers had different strategies, we could not clearly conclude which strategy was better. In fact, in a dart throwing game, the target is not only the bull’s-eye, but some places where the location is significantly higher or lower than the bull’s-eye, e.g., double or triple rings. Therefore, in prospective studies, it is worth investigating which strategy of experts is better to adapt to new targets and how kinematic parameters associated with the strategy change as the target change.

## Conclusions

In summary, we confirmed the two strategies of experts in dart throwing, namely, (1) reducing the timing sensitivity and (2) reducing timing error. However, we used more intuitive methodology, by separately capturing both the kinematics of the projectile and the hand trajectory and analyzing these parameters. We indicated that the two strategies reflected the skill of spatial or timing control along the hand trajectory before the release moment, by the peak value and time of the derived error curve relative to the time of release. In addition, the dart release speed and wrist angular acceleration were the two kinematic parameters which determined the throwing strategy of experts; in other words, it was not simply the variability of the hand’s path, as reported in previous studies.

## Supporting information

S1 File Data. (XLSX)
